# Establishment of an Efficient Primary Culture System for Human Hair Follicle Stem Cells Using the Rho-Associated Protein Kinase Inhibitor Y-27632

**DOI:** 10.3389/fcell.2021.632882

**Published:** 2021-03-05

**Authors:** Lihong Wen, Yong Miao, Zhexiang Fan, Jiarui Zhang, Yixuan Guo, Damao Dai, Junfei Huang, Zhen Liu, Ruosi Chen, Zhiqi Hu

**Affiliations:** ^1^Department of Plastic and Aesthetic Surgery, Nanfang Hospital, Southern Medical University, Guangzhou, China; ^2^Department of Plastic and Burn Surgery, Shenzhen Hospital, Southern Medical University, Shenzhen, China

**Keywords:** human hair follicle stem cells, hair follicle tissue engineering, Y-27632, primary culture system, ERK/MAPK pathway

## Abstract

**Background:**

Hair follicle tissue engineering is a promising strategy for treating hair loss. Human hair follicle stem cells (hHFSCs), which play a key role in the hair cycle, have potential applications in regenerative medicine. However, previous studies did not achieve efficient hHFSC expansion *in vitro* using feeder cells. Therefore, there is a need to develop an efficient primary culture system for the expansion and maintenance of hHFSCs.

**Methods:**

The hHFSCs were obtained by two-step proteolytic digestion combined with microscopy. The cell culture dishes were coated with human fibronectin and inoculated with hHFSCs. The hHFSCs were harvested using a differential enrichment procedure. The effect of Rho-associated protein kinase (ROCK) inhibitor Y-27632, supplemented in keratinocyte serum-free medium (K-SFM), on adhesion, proliferation, and stemness of hHFSCs and the underlying molecular mechanisms were evaluated.

**Results:**

The hHFSCs cultured in K-SFM, supplemented with Y-27632, exhibited enhanced adhesion and proliferation. Additionally, Y-27632 treatment maintained the stemness of hHFSCs and promoted the ability of hHFSCs to regenerate hair follicles *in vivo*. However, Y-27632-induced proliferation and stemness in hHFSCs were conditional and reversible. Furthermore, Y-27632 maintained propagation and stemness of hHFSCs through the ERK/MAPK pathway.

**Conclusion:**

An efficient short-term culture system for primary hHFSCs was successfully established using human fibronectin and the ROCK inhibitor Y-27632, which promoted the proliferation, maintained the stemness of hHFSCs and promoted the ability to regenerate hair follicles *in vivo*. The xenofree culturing method used in this study provided a large number of high-quality seed cells, which have applications in hair follicle tissue engineering and stem cell therapy.

## Introduction

Alopecia results from various factors that decrease the regeneration ability of hair follicles and disrupt the hair cycle (York et al., 2020). Hair transplantation is one of the treatment strategies for hair loss. However, hair transplantation does not provide satisfactory therapeutic outcomes for large-scale hair loss (Vasserot et al., 2019). Although surgical procedures can redistribute hair follicles, they cannot reconstruct the hair follicles. Recent advances in hair tissue engineering have enabled effective treatment of hair loss, especially in cases of large-scale hair loss with insufficient donor hair follicles (Coelho et al., 2012; Owczarczyk-Saczonek et al., 2018). A prerequisite for efficient hair tissue engineering is the availability of seed cell source (Mistriotis and Andreadis, 2013).

Hair follicle stem cells (HFSCs), which are located at the base of the upper permanent portion of the follicular outer root sheath, are involved in mediating the different stages of the hair cycle, namely catagen, telogen, and anagen stages (Ohyama et al., 2006; Jimenez et al., 2011; Myung and Ito, 2012). In the human hair follicles, the HFSCs are characterized by the expression of various immunohistochemical markers, including cytokeratin-15 (CK15), cytokeratin-19 (CK19), and CD200 (Owczarczyk-Saczonek et al., 2018). HFSCs, which are located in easily accessible locations of the human body, exhibit innate characteristics of complete self-renewal and multipotency (ability to differentiate into various lineages) (Myung et al., 2009a,b; Yang et al., 2017). The important characteristic makes HFSCs valuable candidates for regenerative medicine beyond hair and skin regeneration, and wound healing (Mistriotis and Andreadis, 2013; Lim et al., 2018).

Several researchers have been culturing stem cells on 3T3 feeder layers, a technique established by Barrandon and Green (1987), for the last several decades. HFSCs, which are quiescent *in vivo*, rapidly proliferate upon *in vitro* culturing on feeder layers. However, the *in vitro* culturing of HFSCs results in the rapid loss of stem cell characteristics (Blanpain et al., 2004). Some studies have defined the culturing conditions that allow the expansion and maintenance of HFSCs (Chacon-Martinez et al., 2017). The primary culturing of HFSCs in the presence of epithelial growth factor is the most common approach to culture pluripotent cells. However, the cells cultured under these conditions have a poor proliferative ability and tend to differentiate after several passages *in vitro*. Only a little studies have focused on culturing HFSCs from human occipital scalp skin. The use of feeder layers and other matrix Matrigel extracted from mouse sarcoma is associated with the risk of transmission of unknown zoonoses and consequently limits clinical application (Limat et al., 1989). Therefore, there is a need to develop a new culture model for propagating human hair follicle stem cells (hHFSCs).

Rho GTPases and their downstream effectors, such as Rho kinases, are involved in cell adhesion, proliferation, migration, differentiation, and apoptosis through the regulation of the cell microenvironment (David et al., 2012). The Rho-associated protein kinase (ROCK) inhibitor Y-27632 is reported to exert diverse effects on cellular behavior by competing with ATP to bind to the Rho kinase adenosine triphosphate (ATP)-binding pocket (Takahara et al., 2003). Y-27632 can enhance the survival of stem cells from a variety of sources and protect the cultured stem cells from death during cell passage (Watanabe et al., 2007; Terunuma et al., 2010; Wen et al., 2018). Recently, Chapman et al. (2010) demonstrated that culturing human keratinocytes in the presence of Y-27632 markedly increased their proliferation and promoted immortalization. Pakzad et al. (2010) suggested the application of Y-27632, an inhibitor of Rho that regulates various cellular functions, including apoptosis and promoting the interaction of stem cells, to culture the pluripotent stem cells.

To the best of our knowledge, only one study has investigated the Y-27632-mediated regulation of HFSCs isolated from mouse vibrissae. Currently, there are no studies that have evaluated the effects of Y-27632 on the behaviors of HFSCs sourced from human occipital scalp skin (An et al., 2018). In contrast to murine HFSCs that are enriched with CD34-positive stem cells, CD34 is not the major stem cell marker in hHFSCs (Trempus et al., 2003). The hHFSCs express several markers, including CK15, CK19, and CD200 (Owczarczyk-Saczonek et al., 2018). Therefore, the studies on mouse HFSCs cannot be translated to the clinic for treating human diseases, due to the difference in the behaviors of hHFSCs and murine HFSCs. Here, we investigated the effects of Y-27632 on the proliferation, stemness maintenance of hHFSCs and ability to regenerate hair follicles *in vivo* and the underlying mechanisms. This study aimed to establish a novel culture model for hHFSCs using a ROCK inhibitor and to examine the potential application of Y-27632 for the efficient expansion of hHFSCs in hair tissue engineering.

## Materials and Methods

### Tissue Specimens

The occipital scalp skin samples were obtained from discarded tissue of healthy adults (20 men and 3 women; aged 22–39 years old) who underwent selective cosmetic surgery. Ethical approval were obtained from the Medical Ethical Committee of Southern Medical University.

### Animals

Female adult (4–6 weeks old) athymic nude mice (Balb/cAJcl-nu) and newborn C57BL/6J mice were purchased from the Experimental Animal Center of Southern Medical University (Guangzhou, China). All animal studies were conducted under the approval of the Animal Care and Use Committee at the International Medical Center to reduce suffering and provide for the full protection of animal welfare.

### Culturing of hHFSCs

The human scalp samples were rinsed thrice with phosphate-buffered saline (PBS) and the bulge of the hair follicle between the isthmus and the upper part of the hair bulb was separated using microscissors under an MZ8 dissecting microscope (Leica Microsystems, Wetzlar, Germany). The isolated bulge tissue was treated with 0.1% Dispase (Invitrogen, Carlsbad, CA, United States) for 45 min following a previously published protocol (Roh et al., 2005; Garza et al., 2011; Call et al., 2018). The epidermis and dermis were separated and the individual hair shafts, surrounding follicles, and surrounding interfollicular epidermis were simultaneously removed. The isolated epidermis was then washed with PBS and treated with 0.025% trypsin (Gibco, Gaithersburg, MD, United States) for 10 min at 37°C. The samples were vortexed and filtered through a 70 μm filter (Corning, Corning, NY, United States). Next, the filtered samples were centrifuged at 200 *g* for 5 min, seeded in six-well plates coated with 10 μg/mL human fibronectin (Sigma-Aldrich, St. Louis, MO, United States), and cultured in defined keratinocyte serum-free medium (K-SFM, Gibco) (Hakala et al., 2009). The hHFSCs were purified from a mixed population of outer root sheath cells using a differential enrichment procedure. Briefly, the suspended cells were plated onto culture dishes and incubated for 15 min. The cells in the culture medium that exhibited delayed attachment were transferred to a new culture dish.

To passage the cells, the cells were digested using 0.025% trypsin and centrifuged at 300 × g for 5 min after the culture reached a confluency of 80%. The cell pellets were suspended in the corresponding medium and plated onto other cell culture dishes.

### Flow Cytometry

Single-cell suspensions were prepared from the cultured cells as described above. The cells were rinsed once with PBS and incubated with anti-ITGα6 (1:200, eBioscience, San Diego, CA, United States) and anti-CD200 (1:200, eBioscience) antibodies for 30 min on ice. Next, the cells were washed twice with fluorescent-activated cell sorting buffer (2% fetal calf serum and 2 mM ethylenediaminetetraacetic acid in PBS), and analyzed using an LSRFortessa (BD Biosciences, San Jose, CA, United States) flow cytometer. The flow cytometric data were analyzed using FlowJo software version 10 (BD Biosciences).

### Cell Adhesion Assay

To each well of the 24-well plates, 10 μg/mL of fibronectin was added and the plates were incubated at 4°C overnight. The plates were washed thrice with PBS. Next, PBS containing 1% bovine serum albumin (BSA, Sigma) was added to each well and the plate was incubated at 37°C for 1 h. The plates were then washed thrice with PBS. The hHFSCs (1 × 10^5^) were seeded in 24-well plates and treated with 0, 5, 10, or 20 μM Y-27632 (Targetmol, Wellesley Hills, MA, United States) in K-SFM at 37°C for 1 h. The medium containing the non-adherent cells was aspirated. The adherent cells were gently washed with PBS, fixed with methyl alcohol (Solarbio, Beijing, China), and stained with crystal violet (Solarbio). Images were captured using a microscope (Axio Zoom V16, Zeiss, Jena, Germany).

### Cytotoxicity Assay

The hHFSCs were cultured in cell culture dishes, as described above, for 24 h. The culture medium was removed and the cells were treated with 0, 5, 10, or 20 μM Y-27632 in fresh K-SFM for 72 h. The cytotoxicity of Y-27632 was examined using the LIVE/DEAD^®^ Viability/Cytotoxicity kit (Invitrogen) according to the manufacturer’s instructions. The images were captured using a fluorescence microscope (IX73 FL, Olympus, Tokyo, Japan).

### Cell Proliferation Assay

The effect of Y-27632 on hHFSC proliferation was examined using the 5-ethynyl-2′-deoxyuridine (EdU) labeling assay. The hHFSCs (2 × 10^5^ cells per well) seeded in a 24-well plate were treated with 0, 5, 10, or 20 μM Y-27632 in K-SFM for 3 days. Next, the cells were treated with EdU (Ribobio Co., Ltd., Guangzhou, China) for 12 h, fixed, and stained with 1X Apollo567 for 30 min in the dark. The cells were counterstained with Hoechst and imaged using a fluorescence microscope (IX73 FL, Olympus) equipped with a camera. The proliferation rate was assessed by counting the proportion of EdU-positive nuclei (red) among the nuclei exhibiting blue fluorescence (six random microscopic fields were evaluated in each well). The data were obtained from five independent experiments.

### Colony Formation Assay

The hHFSCs (1 × 10^4^ cells per well) seeded in six-well plates were stimulated with 0, 5, 10, or 20 μM Y-27632 for 5 days. The cells were fixed in 4% paraformaldehyde (Solarbio) for 30 min. The total number of colonies was counted after staining with crystal violet for 15 min. The ability of hHFSCs, subjected to different treatments, to form colonies (groups of 50 or more adhering cells derived from the same mother cell) was assessed.

### Immunocytochemistry and Immunohistochemistry

The cells were seeded (1 × 10^4^ cells per well) in 24-well plates and cultured until approximately 30% confluency. Next, the cells were fixed in 4% paraformaldehyde and permeabilized with 0.1% Triton X-100 (Solarbio) in PBS for 10 min at room temperature. Further, the cells were blocked with 3% BSA in PBS and incubated with anti-CK15 (1:200, Abcam) and anti-CK19 (1:200, Abcam) antibodies in 1% BSA overnight at 4°C, followed by incubation with Cy3 anti-mouse secondary antibodies (1:500, Beyotime Institute of Biotechnology, Jiangsu, China) or Alexa Fluor^®^ 488-labeled anti-rabbit secondary antibodies (1:500, Abcam) in the dark for 1 h at room temperature. The nuclei were stained with 4, 6-diamino-2-phenylindole (1:200, eBioscience). The images were captured using a fluorescent microscope (BX63, Olympus) equipped with a digital camera. The negative control comprised cells incubated with secondary antibody alone. The consecutive human scalp sections were immunohistochemically stained with anti-cytokeratin 15 (1:50, Abcam) antibody, following a previously described protocol (Inoue et al., 2009).

### Histochemical Staining

The explanted hair follicles with attached surrounding tissue were fixed in 10% buffered formalin and embedded in paraffin wax. Next, the 5 μm thick sections were stained with hematoxylin and eosin (Solarbio) following standard procedures for visual identification and assessment of structural integrity of hair follicles, using a microscope (BX51, Olympus).

### Quantitative Real-Time Polymerase Chain Reaction (qRT-PCR)

Total RNA was extracted from the cells subjected to different treatments, using Trizol (Takara, Tokyo, Japan). The mRNA was reverse-transcribed to complementary DNA (cDNA) using the PrimeScript RT-PCR kit (Takara) according to the manufacturer’s s instructions. The qRT-PCR analysis was performed with SYBR Premix Ex Taq II (Tli RNaseH Plus; Takara) in a Light Cycle Roche 480 II Real-time PCR system (Roche, Basel Switzerland) in triplicate. The primer sequences used for qRT-PCR analysis are listed in Table 1. The expression levels of target genes were normalized to those of *GAPDH*. The relative expression level was calculated using the 2^–ΔΔ*Ct*^ method.

**TABLE 1:** Primer sequences for qRT-PCR.

**Gene**	**Forward primer (5′–3′)**	**Reverse primer (5′–3′)**
GAPDH	GCACCGTCAAGGCTGAGAAC	TGGTGAAGACGCCAGTGGA
CK15	GACGGAGATCACAGACCTGAG	CTCCAGCCGTGTCTTTATGTC
CK19	AACGGCGAGCTAGAGGTGA	GGATGGTCGTGTAGTAGTGGC
Nanog	CAGAAGGCCTCACACCTAC	ATTGTTCCAGGTCTGGTTGC
Oct4	CACTGTACTCCTCGGTCCCTTTC	CAGGCACCTCAGTTTGAATGC

### Western Blotting

Total proteins were extracted from cells using radioimmunoprecipitation assay (RIPA) lysis buffer (Beyotime, Shanghai, China) supplemented with protease and phosphatase inhibitor cocktail (Roche), following the manufacturer’s instructions. The protein concentration was determined using the bicinchoninic acid protein assay kit (Thermo Scientific). The protein sample mixed with sodium dodecyl sulfate-polyacrylamide gel electrophoresis (SDS-PAGE) loading buffer was subjected to denaturation at 100°C for 5 min. Equal amounts of proteins (20 μg) were subjected to SDS-PAGE. The resolved proteins were transferred to a polyvinylidene fluoride membrane (Millipore). The membrane was blocked with non-fat dry milk for 1 h and incubated with the following primary antibodies overnight at 4°C: mouse anti-CK15 (1:1,000; Abcam), rabbit anti-CK19 (1:1,000; Abcam), rabbit anti-ERK1/2 (1:1,000; Cell Signaling Technology), rabbit anti-phospho-ERK1/2 (1:1,000; Cell Signaling Technology), and anti-GAPDH (1:10,000; Proteintech) antibodies. Next, the membrane was incubated with horseradish peroxidase-labeled secondary antibodies (1:2,000; Beyotime) for 1 h at room temperature. The membrane was washed thrice with tris-buffered saline containing 1% Tween-20. The immunoreactive bands were visualized using enhanced chemiluminescence reagents (Millipore). The level of target proteins was normalized to that of GAPDH before statistical analysis. The Image J 1.44 software (NIH, Bethesda, Maryland, United States) was used to quantify protein expression.

### *In vivo* Hair Regeneration

Neonatal epidermal cells and dermal cells were prepared as described previously (Xiao et al., 2016). Briefly, the trunk skin of neonatal C57BL/6J mice was mechanically separated and digested with 0.1% Dispase at 37°C for 1 h. The skin specimen was then divided into epidermis and dermis using forceps. The epidermis was minced and digested in 0.025% trypsin at 37°C for 10 min and the dermis was minced and digested in 0.2% collagenase (Sigma-Aldrich, St. Louis, MO, United States) at 37°C for 1 h. After digestion, an equal volume of 10% FBS in DMEM was added to terminate the reaction, and the samples were filtered through 70 μm strainers. Following centrifugation and washing, murine neonatal epidermal cells and dermal cells were obtained.

For *in vivo* implantation, five groups were arranged for this experiment as (1) pre-prepared murine neonatal dermal cells alone (as negative control), (2) a mixture of murine neonatal epidermal cells and murine neonatal dermal cells (as positive control), (3 and 4) a mixture of P1 hHFSCs with or without 10 μM Y-27632 and pre-prepared murine neonatal dermal cells and (5) a mixture of P3 hHFSCs with 10 μM Y-27632 and pre-prepared murine neonatal dermal cells. Unless otherwise stated for each intracutaneous injection, 1 × 10^6^ dermal cells and 5 × 10^5^ epidermal cells or hHFSCs were resuspended (50 μL of DMEM) and injected (29-gauge needle, BD Biosciences) into the hypodermis of nude mice, forming a bleb. After 3 weeks, the skin at the injection spot was dissected. The number of hair follicles formed was quantified by microscopic photography and morphometry. The hair reconstruction by hHFSCs was confirmed in Paraffin section via immunohistochemically stained by the human-specific marker (anti-HLAA, 1:100, Abcam).

### Statistical Analysis

All statistical analyses were performed using the GraphPad Prism 8 software (GraphPad Software, Inc., La Jolla, CA, United States). The data are expressed as mean ± standard deviation. Data from each experimental condition were analyzed for normality using the Kolmogorov–Smirnov test. The data were analyzed using the Wilcoxon test and *t*-test. The difference was considered significant at p < 0.05. Each experiment was repeated at least three times.

## Results

### Primary Culture of hHFSCs From the Occipital Scalp Skin

**FIGURE 1:**
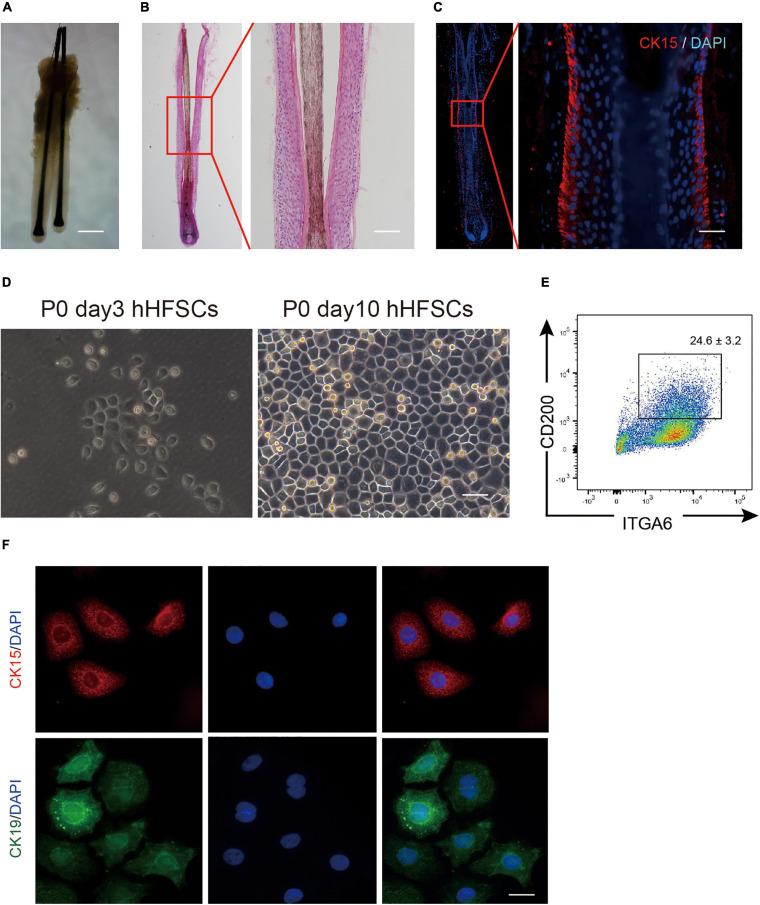
Isolation and cultivation of hHFSCs. **(A)** Morphology of intact human hair follicles from the occipital scalp skin. Scale bars: 200 μm. **(B)** Morphology of an intact human hair follicle examined using H&E staining. Scale bars: 100 μm. **(C)** Section immunostained with anti-CK15 antibody showing the biological bulge zone. Scale bars: 50 μm. **(D)** Morphology of P0 hHFSCs cultured for 3 and 10 days. Scale bars: 100 μm. **(E)** Representative flow cytometry plots of P0 hHFSCs cultured in K-SFM. **(F)** Immunofluorescence analysis of CK15 and CK19 in the attached hHFSCs. Scale bars: 25 μm. All data are presented as mean ± standard deviation from at least three independent experiments. hHFSCs, human hair follicle stem cells; H&E, hematoxylin and eosin, K-SFM, keratinocyte serum-free medium.

The bulge zone of human hair follicles from the occipital scalp skin was identified based on CK15 and CK19 immunoreactivities. As described previously, the bulge zone was identified below the entrance of the sebaceous duct into the follicle at an average depth of 1 mm below the skin surface extending to a depth of 1.8 mm just below the arrector pili muscle insertion (Oh et al., 2011; Figures 1A–C). Differential interference contrast microscopy and histological examinations revealed that the plucked human hair follicles were intact with minimal dermal contamination. The follicular epithelium was trypsinized and filtered to obtain a single-cell suspension containing cells of varying sizes and shapes. The dissociated single cells were harvested using centrifugation, cultured in K-SFM, and purified using a differential enrichment procedure. After 3 days, the pre-attached cells appeared as clustered colonies comprising cells with keratinocyte-like morphology with a large nucleus and a high nuclear-to-cytoplasmic ratio (Figure 1D). The P0 hHFSCs from occipital scalp skin comprised 24.6 ± 3.2% CD200^+^ ITGα 6^+^ HFSCs (Figure 1E). Immunofluorescence staining identified the stem cell markers CK15 and CK19 in hHFSCs (Figure 1F).

### Y-27632 Promotes Cell Adhesion of hHFSCs in Primary Culture

**FIGURE 2:**
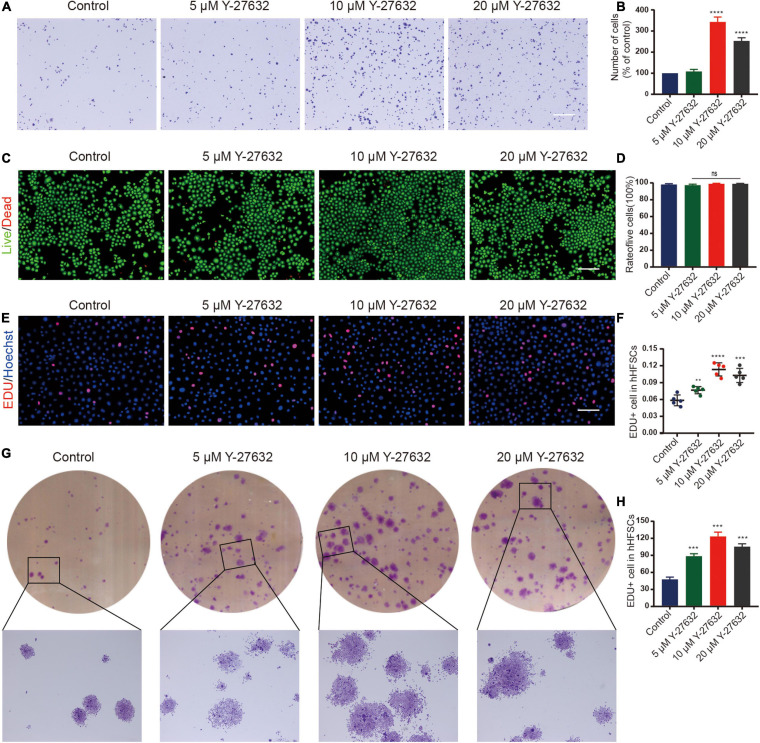
Y-27632 promoted the adhesion and proliferation of hHFSCs. **(A,B)** The hHFSCs treated with 0, 5, 10, or 20 μM of Y-27632 were subjected to cell adhesion assay. Scale bars: 250 μm. **(C)** Live/dead staining of hHFSCs on day 5 of culturing. Live and dead cells are shown in green and red, respectively; Scale bars: 100 μm. **(D)** The proportion of live hHFSCs was not significantly different among the treatment groups (*p* > 0.05). **(E)** The hHFSCs treated with or without Y-27632 were subjected to the EdU labeling assay [EdU-positive (red); Hoechst (blue)]. Scale bars: 50 μm. **(F)** The proportion of EdU-positive cells in the Y-27632-treated group was significantly higher than that in the control group. **(G,H)** Colony formation assays further confirmed that the proliferation of hHFSCs was significantly upregulated upon treatment with Y-27632. All data are presented as mean ± standard deviation from at least three independent experiments. Student’s *t*-test. ns, not significant; hHFSCs, human hair follicle stem cells; EdU, 5-ethynyl-2′-deoxyuridine. **p < 0.01, ***p < 0.001, and ****p < 0.0001.

Most single cells did not adhere to the plate and gradually died at the initial stages of the primary culture of hHFSCs. A few cells attached to the plate and continued to grow slowly as a monolayer. To investigate the effect of Y-27632 on the adhesion of hHFSCs, P0 hHFSCs were cultured in K-SFM supplemented with 0, 5, 10, or 20 μM Y-27632, for 1 h. The results of the cell adhesion assay indicated that Y-27632 dose-dependently promoted the adhesion of hHFSCs. The adhesion of hHFSCs treated with 10 and 20 μM of Y-27632 was not significantly different. However, the adhesion of hHFSCs treated with 10 μM Y-27632 was higher than that of hHFSCs treated with 5 μM Y-27632 (Figures 2A,B).

### Y-27632 Promoted the Proliferation of hHFSCs

The cytotoxicity of different concentrations of Y-27632 against hHFSCs was determined using the LIVE/DEAD kit. Treatment with 0, 5, 10, or 20 μM of Y-27632 did not significantly affect the viability of hHFSCs (Figures 2C,D). The results of the EdU labeling assay revealed that the proportion of EdU-positive cells in the hHFSCs treated with 5, 10, and 20 μM Y-27632 (7.65 ± 0.63, 11.35 ± 1.17, and 10.28 ± 1.26%, respectively) was significantly higher than that in the negative control group (5.84 ± 0.96%; p < 0.01). The optimal concentrations of Y-27632 to promote hHFSC proliferation were 10 and 20 μM (Figures 2E,F).

Treatment with Y-27632 significantly increased the number of colony-forming units of hHFSCs (>50 cells/colony). Additionally, the colonies exhibited an enlarged morphology (>100 cells/colony) (Figures 2G,H). The number of colonies derived from cells treated with 10 μM Y-27632 (123.30 ± 7.57 colonies/well) was not significantly different from that derived from cells treated with 20 μM Y-27632 (115.30 ± 5.03 colonies/well) (*p* > 0.001). This indicated that 10 and 20 μM Y-27632 significantly promoted the proliferation of hHFSCs. For further experiments, 10 μM Y-27632 was used.

### Y-27632 Maintained the Stemness of hHFSCs

**FIGURE 3:**
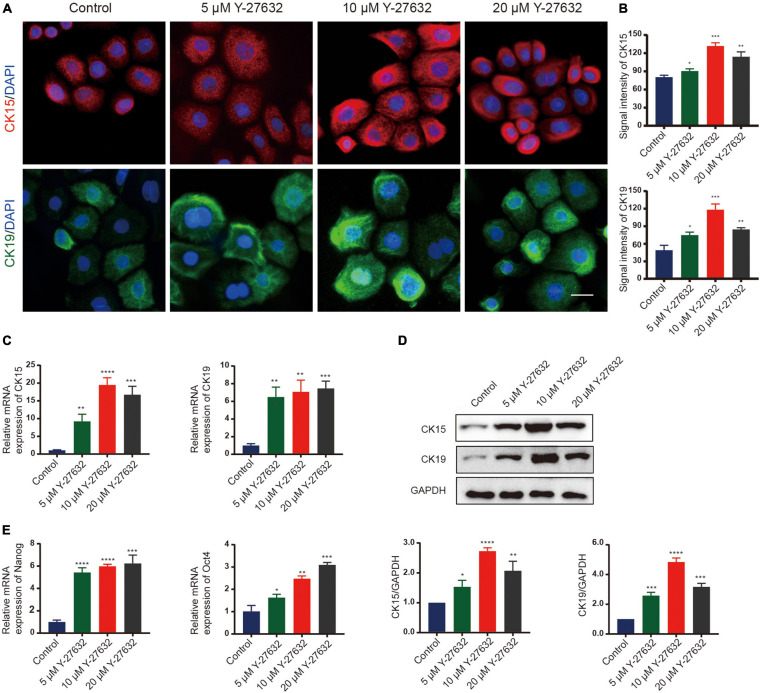
Y-27632 maintained the stemness of hHFSCs. **(A)** Immunofluorescence staining of P1 hHFSCs was performed on day 5 of culturing. Scale bars: 25 μm. **(B)** The signal intensities of CK15 and CK19 in hHFSCs in the Y-27632-treated group were significantly higher than those in the control group. **(C)** Y-27632 upregulated the expression levels of CK15 and CK19 after 5 days of treatment. **(D)** Western blotting analysis of CK15 and CK19 expression levels. **(E)** The qRT-PCR analysis of Nanog and Oct4 mRNA expression. The qRT-PCR results are shown as fold-change relative to the expression in Y-27632-treated hHFSCs. hHFSCs, human hair follicle stem cells; qRT-PCR, quantitative real-time polymerase chain reaction. *p < 0.05, **p < 0.01, ***p < 0.001, and ****p < 0.0001.

The effect of Y-27632 on the stemness of hHFSCs was analyzed by examining the two stemness markers using immunocytochemistry. Compared with the control group, treatment with 5, 10, and 20 μM Y-27632 significantly increased the number of CK15-labeled hHFSCs (*p* < 0.01). Furthermore, the expression of CK15 in the cells treated with 10 μM and 20 μM Y-27632 was higher than that in the control group (Figures 3A,B). The expression of markers in hHFSCs was also detected using qRT-PCR and western blotting. The expression levels of CK15 and CK19 in the cells treated with Y-27632 were significantly higher than those in the control group (Figure 3C). The results of qRT-PCR analysis were consistent with those of western blotting (Figure 3D). Nanog and Oct4 are reported to contribute to the self-renewal of stem cells (Yu et al., 2006; Liu et al., 2019; Lu et al., 2019). The qRT-PCR analysis showed that the expression levels of Nanog and Oct4 in the cells treated with Y-27632 were significantly higher than those in the control group (Figure 3E).

### Effect of Y-27632 on hHFSCs Was Conditional

**FIGURE 4:**
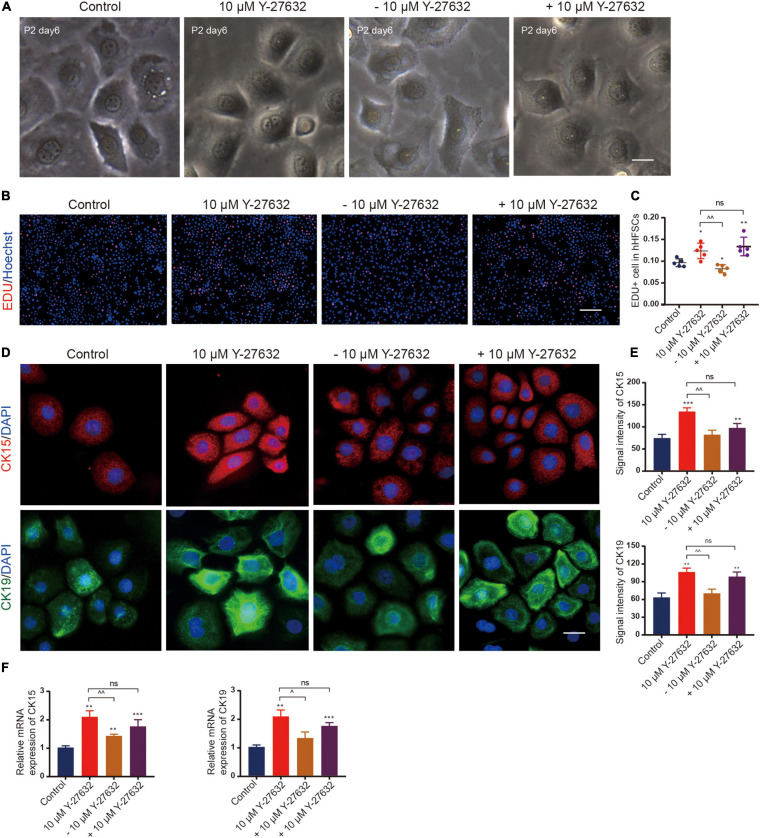
The effect of Y-27632 on hHFSCs was conditional. **(A)** Morphology of P2 hHFSCs cultured for 6 days with or without Y-27632. Scale bars: 25 μm. **(B)** The hHFSCs treated with or without Y-27632 were subjected to the EdU labeling assay [EdU-positive (red); Hoechst (blue)]. Scale bars: 100 μm. **(C)** The proportion of EdU-positive cells in the Y-27632-treated group was significantly higher than that in the control group or Y-27632 withdrawal group. **(D)** Immunofluorescence staining of P2 hHFSCs was performed on day 5 of culturing. Scale bars: 50 μm. **(E)** The signal intensities of CK15 and CK19 in the Y-27632-treated group were significantly higher than those in the control group or Y-27632 withdraw group. **(F)** The qRT-PCR analysis of CK15 and CK19 mRNA expression. ns, not significant; hHFSCs, human hair follicle stem cells; EdU, 5-ethynyl-2′-deoxyuridine; qRT-PCR, quantitative real-time polymerase chain reaction. *compared with the control group; ^Y-27632-treated group vs. other experimental groups. *p < 0.1, **p < 0.01, ***p < 0.001, ^*p* < 0.1, ^^*p* < 0.01, and ^^^*p* < 0.001.

Next, the effect of continuous ROCK inhibition on the proliferation of hHFSCs was examined. The cells were cultured in the presence of Y-27632 for 3 days and the inhibitor was removed thereafter. In the absence of Y-27632, the hHFSCs exhibited a delayed growth and reached senescence quickly, as evidenced by the disintegration of the nuclear membrane. However, the hHFSCs treated with 10 μM Y-27632 exhibited logarithmic growth (Figure 4A). The EdU labeling assay was performed to detect the effect of Y-27632 removal from the culture medium on the proliferation of hHFSCs. The proportion of EdU-positive cells among the cells cultured in the absence of Y-27632 was lower than that among the cells cultured in the presence of 10 μM Y-27632 (Figures 4B,C). Immunocytochemistry analysis was performed 3 days after the removal of Y-27632 to examine the expression of stemness markers of hHFSCs. The expression levels of CK15 and CK19 were downregulated in the clustered colonies at 72 h post-Y-27632 withdrawal (Figures 4D,E).

Conversely, Y-27632 was added to hHFSCs that had been cultured for 3 days in the absence of Y-27632. The rate of proliferation increased immediately even in the cells that were only a few cell divisions from reaching senescence (Figures 4B,C). The expression levels of CK15 and CK19 were also upregulated (Figure 4F). These results suggest that ROCK inhibition is necessary for the propagation and maintenance of hHFSCs *in vitro*.

### Y-27632 Maintained hHFSCs Propagation and Stemness Through the ERK Signaling Pathway

**FIGURE 5:**
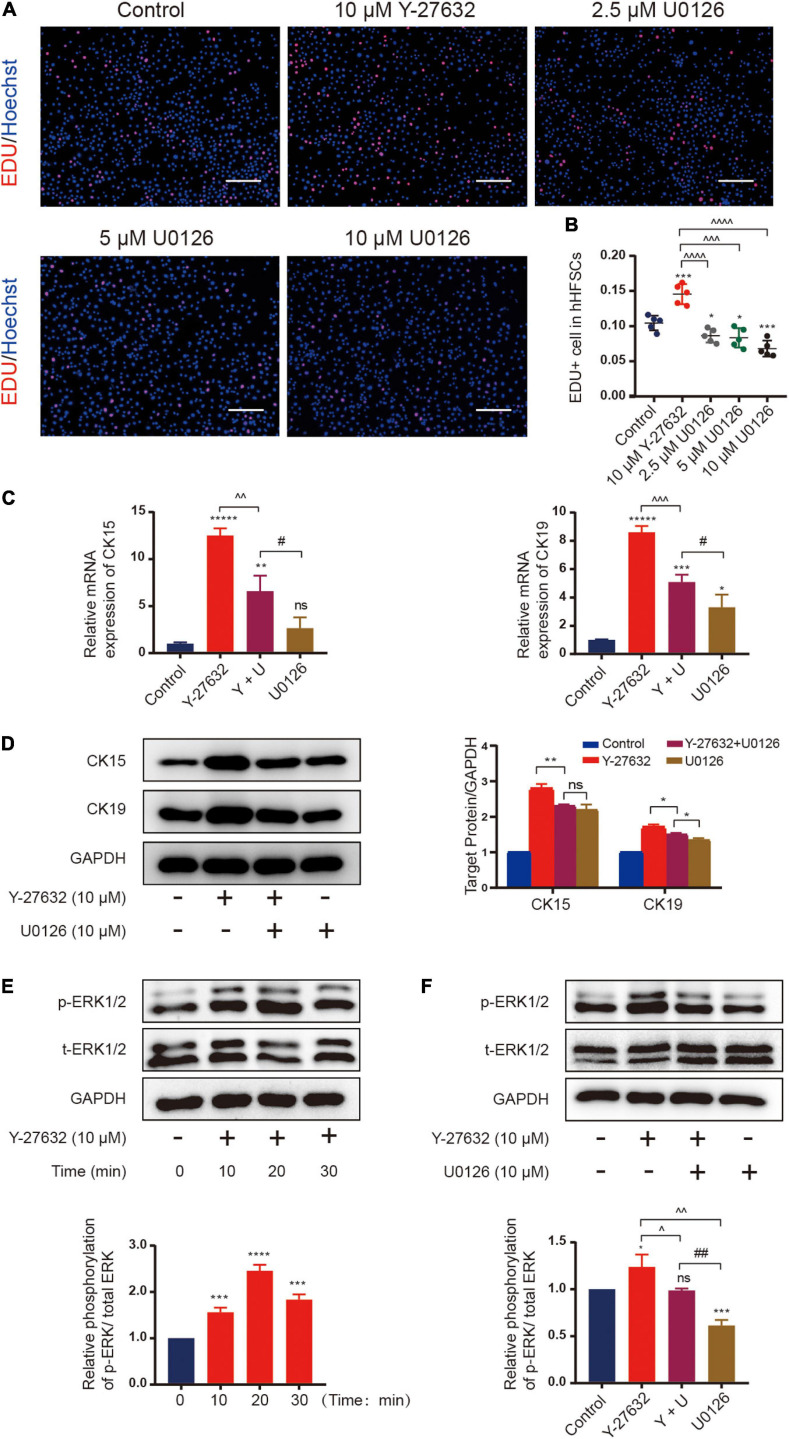
Y-27632 maintained hHFSCs propagation and stemness through the ERK signaling pathway. **(A)** The hHFSCs treated with Y-27632 (10 μM) and 2.5, 5, or 10 μM U0126 (ERK inhibitor) for 72 h were subjected to the EdU labeling assay. Scale bars: 100 μm. **(B)** The proportion of EdU-positive cells significantly decreased upon treatment with U0126. **(C)** The increased mRNA expression of CK15 and CK19 induced by 10 μM Y-27632 was blocked by 10 μM U0126. **(D)** Western blotting analysis of CK15 and CK19 expression levels. **(E)** The hHFSCs were treated with 10 μM Y-27632 for 0, 10, 20, and 30 min and subjected to western blotting analysis to examine the level of p-ERK. **(F)** The p-ERK level was evaluated in the presence or absence of 10 μM U0126. U0126 attenuated Y-27632-induced ERK activity. hHFSCs, human hair follicle stem cells; EdU, 5-ethynyl-2′-deoxyuridine; qRT-PCR, quantitative real-time polymerase chain reaction. Data are presented as the mean ± standard error of mean from three independent experiments. *Compared with the control group; ^Y-27632-treated group vs. other experimental groups; #U0126-treated group vs. other experimental groups. *p < 0.05, **p < 0.01, ***p < 0.001, ****p < 0.0001, *****p < 0.00001, ^*p* < 0.1, ^^*p* < 0.01, ^^^*p* < 0.001, ^^^^*p* < 0.0001, and ^##^*p* < 0.01.

Next, we examined the signaling pathways that mediate the mechanisms underlying Y-27632-induced cell proliferation and stemness maintenance. The mitogen-activated protein kinase (MAPK) cascades are central signaling elements that regulate cell proliferation and differentiation. The Y-27632-induced proliferation of hHFSCs was significantly inhibited upon treatment with 2.5, 5, or 10 μM of U0126, an inhibitor of extracellular signal-regulated kinase 1/2 (ERK) (Figures 5A,B). Then, we tested whether 10 μM of U0126 could downregulated the expression of stemness marker induced by Y-27632 on hHFSCs using qRT-PCR and western blotting. The results showed that the increased CK15 and CK19 expression induced by Y-27632 was blocked by U0126 (Figures 5C–E).

The mechanism underneath on the effect of Y-27632 was examined using western blotting. The hHFSCs were stimulated with 10 μM Y-27632 for 0, 10, 20, and 30 min. Y-27632 upregulated ERK phosphorylation at 10 min post-stimulation with a peak level at 20 min and gradually downregulated ERK phosphorylation thereafter (Figure 5F). The effect of U0126 on Y-27632-induced ERK activity was examined by evaluating ERK phosphorylation in the presence or absence of 10 μM U0126. Western blotting analysis revealed that the phosphorylation level of ERK decreased upon treatment with 10 μM U0126 (Figure 5G). These results indicate that the ERK/MAPK signaling pathways mediate Y-27632-induced hHFSC proliferation and stemness maintenance.

### Y-27632 Promoted hHFSCs on the Capability of Regenerating Hair Follicles *in vivo*

**FIGURE 6:**
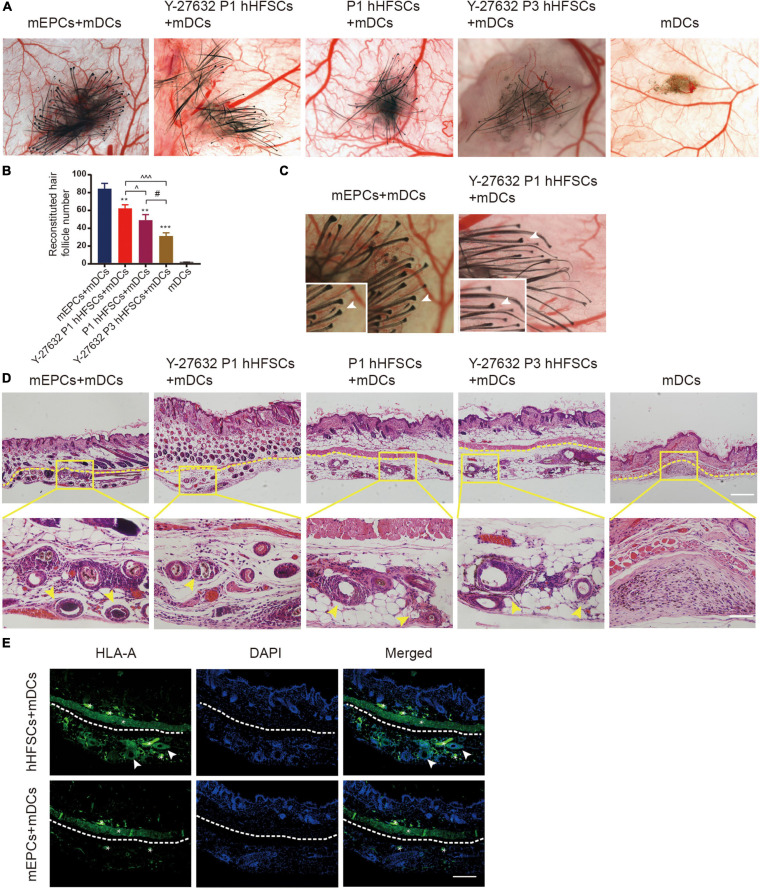
Y-27632 promoted hHFSCs on the capability of regenerating hair follicles *in vivo*. **(A)** Stereoscopic images of recipient sites after 3 weeks post-transplant. **(B)** Analysis of the reconstituted hair follicle number among the negative group, the positive group and experimental groups. **(C)** The morphology of the *de novo* hair follicles were intact. **(D)** Histochemical staining of reconsitituted skin (the yellow dotted line divides the injection area and mouse skin) exhibited a large number of mature HFs in the positive group and experimental groups. Scale bars: 100 μm. **(E)** Immunohistochemically staining showed positive expression of human-specific marker in the hair follicle cells differentiated by hHFSCs. *non-specificity staining. Scale bars: 100 μm. mEPCs, murine neonatal epidermal cells; mDCs, murine neonatal dermal cells; hHFSCs, human hair follicle stem cells. *compared with the positive control group; ^Y-27632-treated P1 group vs. other experimental groups; ^#^Y-27632-treated P3 group vs. other experimental groups. **p < 0.01, ***p < 0.001, ^*p* < 0.1, ^^^*p* < 0.001, ^#^*p* < 0.1.

We further performed functional analysis on the hHFSCs with Y-27632 using a reconstitution assay, which tests the ability of hHFSCs to regenerate hair follicles. We observed that the negative control groups which were only grafted with neonatal dermal cells did not exhibit new hair formation. Similar to positive control groups, *de novo* hair shafts were induced in recipient sites after 3 weeks of implantation in experimental groups which were grafted with a mixture of hHFSCs with or without Y-27632 and neonatal dermal cells. From stereoscopic observation, both P1 hHFSCs cultured in general medium and P1 hHFSCs treated with Y-27632 could induced hair follicles (Figure 6A). The morphology of the *de novo* hair follicles were intact, including the dermal papilla, matrix cells and other epithelial tissue (Figure 6C). Obviously, significantly more hair follicles formed in the group treated with Y-27632 than those in the control group (Figures 6A,B). Furthermore, Y-27632 maintained the multipotency of hHFSCs after several passages. The results showed that P3 hHFSCs with Y-27632 can also displayed an ideal HF inductivity (Figures 6A,B).

To investigate the internal structures of the reconsitituted skin, we performed tissue sectioning and HE staining. Histologic sectioning exhibited that a large number of mature HFs distributed within the hypodermic layer in the positive group and experimental groups (Figure 6D). Immunohistochemically staining showed positive expression of human-specific marker in the hair follicle cells differentiated by hHFSCs (Figure 6E).

## Discussion

**SCHEME 1:**
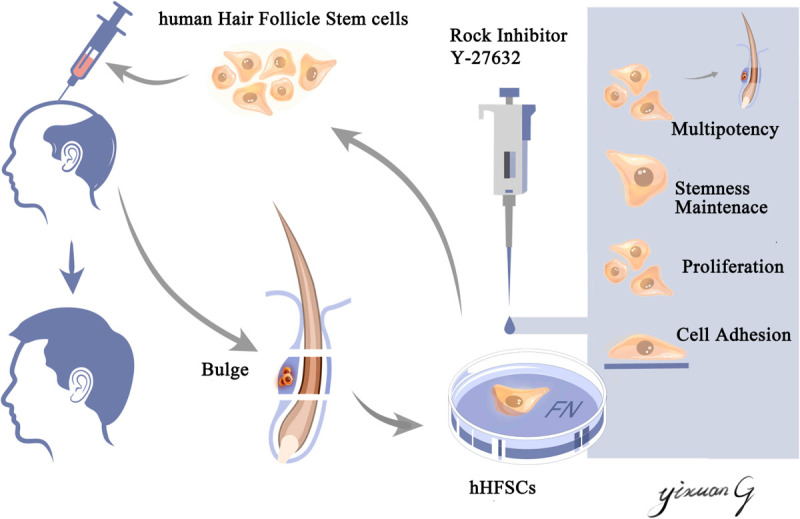
Schematic illustration of an efficient primary culture system for human hair follicle stem cells isolated from occipital scalp skin using human fibronectin and ROCK inhibitor Y-27632. This culture model facilitated the proliferation, maintained the stemness of human hair follicle stem cells and the capability of regenerating hair follicles *in vivo*. This culture model may aid in obtaining an increased number of high-quality seed cells, which have applications in hair follicle tissue engineering and stem cell therapy.

An ideal stem cell culture system should preserve the viability, promote proliferation, and maintain the stemness of hHFSCs, which are the major requirements for obtaining high-quality seed cells in hair follicle tissue engineering. In this study, we successfully established a xenofree primary culture system for hHFSCs. The hHFSCs cultured in K-SFM supplemented with Y-27632 exhibited enhanced adhesion and proliferation. Moreover, Y-27632 maintained the stemness of hHFSCs and the ability of hHFSCs to regenerate hair follicles *in vivo*. The Y-27632-induced proliferation and stemness in hHFSCs were conditional and reversible. Furthermore, Y-27632 maintained hHFSCs propagation and stemness through the ERK/MAPK pathway (Scheme 1).

The hHFSCs have potential applications in hair follicle regenerative medicine, as they can undergo complete self-renewal and exhibit multipotency (differentiate into various lineages) (Yang et al., 2017). However, an efficient *in vitro* primary culture system for hHFSCs is currently not established. The main factors affecting the proliferation and biological characteristics of an adherent culture *in vitro* are the coated culture dishes and culture medium (Hong et al., 2019; Hashimoto et al., 2020). In this study, we obtained hHFSCs using a two-step proteolytic digestion combined with microscopy. The culture dishes were coated with an animal origin-free coating matrix, containing the human extracellular matrix, before culturing hHFSCs. The hHFSCs were then purified using a differential enrichment procedure, which utilizes the delayed adhesion of hHFSCs to the surface of a culture dish. Compared with the 3T3 feeder cells, mouse collagen type IV, and basement membrane matrix Matrigel extracted from mouse sarcoma in the traditional culture system, human fibronectin can prevent the transmission of unknown zoonoses, which is critical for the clinical application of hHFSCs (Limat et al., 1989; Li et al., 2015; Call et al., 2018).

The use of K-SFM, which was optimized for the isolation and expansion of hHFSCs, prevents the potential contamination derived from fibroblasts. However, the expression of CK15 was rapidly downregulated upon culturing the hHFSCs in K-SFM for a prolonged period (Blanpain et al., 2004). Some studies have suggested that the addition of serum may prevent the downregulation of CK15. However, the use of serum may contribute to the transmission of unknown zoonoses and the induction of immunological reactions after clinical transplantation (Limat et al., 1989). The small molecule Y-27632 can reprogram and modulate the state of stem cells and is considered as one of the alternatives to serum and macromolecular proteins for *in vitro* culturing (Li et al., 2015).

Y-27632, an inhibitor of ROCK, modulates various cellular functions, including actin cytoskeleton organization, cell adhesion, cell motility, and apoptosis (David et al., 2012). Additionally, Y-27632, which was developed as an inhibitor of the calcium-sensitization pathway involved in smooth muscle contraction, inhibits RhoA-induced formation of stress fibers and focal adhesions (Uehata et al., 1997). Watanabe et al. (2007) first applied Y-27632 to inhibit apoptosis of dissociated single human embryonic stem cells in adherent cultures. Treatment with Y-27632 enhanced the adhesion and survival of human embryonic stem cells in adherent cultures during passage, by inducing cytoskeletal changes. Consistent with these results, this study demonstrated that only a few cells attached to the plate and slowly formed a monolayer in the primary culture of hHFSCs. Most cells underwent apoptosis as they did not attach to the culture plate. The dissociated single cells exhibited enhanced survival upon treatment with Y-27632. Moreover, Y-27632 dose-dependently increased the adhesion of hHFSCs.

The hallmarks of hHFSCs are enhanced proliferation and multipotency. The supplementation of Y-27632 can regulate the ability of stem cells to self-renew and differentiate into derivatives of all three germ layers (Sivasubramaiyan et al., 2009; Kurosawa, 2012). The use of Y-27632 during primary cultures offers a simple and effective way to prepare a large number of human epithelial stem cells from skin tissues (Terunuma et al., 2010; Wen et al., 2018). In this study, 10 and 20 μM Y-27632 significantly promoted the proliferation of primary hHFSCs. The challenges associated with *in vitro* culture of hHFSCs are low adhesion ratio and poor proliferation during passaging. The colony formation rate is an important indicator for evaluating the reattachment rate and the proliferation ability of hHFSCs. The number of clones directly affects the scale of cell expansion. In this study, the hHFSC colonies in K-SFM supplemented with Y-27632 were larger and more clustered than those in the control groups. These results suggest that Y-27632 facilitates the proliferation of hHFSCs.

Passage is one of the important indicators for evaluating the effectiveness of culture system. Y-27632 promotes the long-term proliferation of primary human keratinocytes and these cells efficiently bypassed senescence and became immortal (Chapman et al., 2010). However, passage and long-term proliferation is still a challenges in the culture of hHFSCs *in vitro* because of the different biological behavior of mouse and human stem cells (Yu et al., 2006; An et al., 2018). In our research, we found that both hHFSCs with or without Y-27632 at early passages (P1-2) were actively dividing and appeared small, cuboidal, and homogeneous. However, less cells could reattached onto culture dishes and became senescence with the flat and heterogeneous morphology at later passages (P3) in the untreated group. The hHFSCs with Y-27632 can be continuously cultured for at least 5 passages with a healthy status. Unfortunately only approximately 50% of the treated cells escaped senescence at passage 6 (Supplementary Figure S1). This data shown indicated that the culturing system we established is suit to short-term culture.

Self-renewal and multipotency (ability to differentiate into various lineages) are innate characteristics of HFSCs (Myung et al., 2009a). The supplementation of Y-27632 in K-SFM significantly upregulated the expression of hHFSC stemness markers (CK15 and CK19) and pluripotent markers (Nanog and Oct4). This was consistent with the results of previous studies, which demonstrated that Y-27632 upregulated Oct-3/4 expression in small colonies of human embryonic stem cells and spatiotemporally altered the balance between pluripotency and early differentiation events (Peerani et al., 2007; Sivasubramaniyan et al., 2010).

Our study further demonstrates the culture of hHFSCs in Y-27632 medium maintained their differentiation potential *in vivo*. *de novo* hair shafts were induced in recipient sites after 3 weeks of implantation with a mixture of hHFSCs and neonatal dermal cells. The culture system with Y-27632 appear to have stabilized the hair potential of the cells. Obviously, nearly 1.5-fold change more hair follicles formed in the group treated with Y-27632, compared with the control group. And we also demonstrated that Y-27632 maintained the multipotency of hHFSCs after several passages. Though there are some evidences that normal keratinocytes isolated from human tissue, for instance, isolated from fetal and newborn human foreskin epidermal cells combined with neonatal murine dermal cells could regenerate hair follicles *in vivo*, it involved the ethics of fetal-derived cells (Wu et al., 2014; Abaci et al., 2018). hHFSCs, which are located in easily accessible locations of human body, exhibit complete self-renewal and ability to differentiate to various lineages. These characteristic makes hHFSCs valuable seeder for hair follicle regeneration.

Chapman et al. (2014) demonstrated that the supplementation of ROCK inhibitor to the keratinocyte culture system promoted indefinite cellular proliferation, which was conditional and reversible. The removal of the ROCK inhibitor delayed cell growth and senescence after a few passages. However, Gao et al. (2019) demonstrated that treatment with Y-27632 enhanced the survival and adhesion of human embryonic stem cells but induced detachment and apoptosis of the attached cells. The authors suggested that the use of Y-27632 should be limited to 24 h to optimize its pro-survival effects. This may be because Y-27632 exerted different effects in different cell types and culture conditions. Therefore, it is important to determine the effect of long-term applications of Y-27632 in the culture medium of hHFSCs. The Y-27632-induced proliferation and stemness maintenance were reversible. The morphology of hHFSCs rapidly changed into a senescence phenotype and they exhibited a flat and heterogeneous morphology with an enlarged cytoplasmic volume after the removal of the ROCK inhibitor. Additionally, the removal of the ROCK inhibitor decreased the proliferation and reversed the stemness of hHFSCs. These results suggest that Y-27632 should be used in both the primary and short-term culture of hHFSCs routinely. This was consistent with the findings of Pakzad et al., who reported that the ROCK inhibitor, which regulates multiple cellular functions, should be routinely used in pluripotent stem cell culture (Pakzad et al., 2010).

Several studies have demonstrated that ROCK inhibitors regulate cell proliferation by activating different growth-regulatory proteins and triggering different signals in different cells (Yu et al., 2012; Wang et al., 2017). The MAPK cascades are central signaling elements that regulate various physiological processes, including cell proliferation, apoptosis, and stress response (Guo et al., 2020). ERK belongs to the MAPK family, which regulates the signaling cascades and transmits extracellular signals to intracellular targets (Garcia-Gomez et al., 2018). Previous studies have demonstrated that the ERK/MAPK signaling pathway is involved in the Y-27632-induced proliferation of periodontal ligament stem cells and Y-27632 maintained the balance of keratinocyte proliferation and differentiation through regulation of ERK activity (Wang et al., 2017; Zhang et al., 2019). Therefore, we examined the signaling pathways to determine the underlying mechanisms of Y-27632-induced proliferation and stemness maintenance of hHFSCs. Y-27632 dose-dependently enhanced cell proliferation within the concentration range of 5–20 μM. Treatment with U0126, an ERK inhibitor, mitigated the Y-27632-induced hHFSC proliferation and downregulated the expression of stemness marker induced by Y-27632. Meanwhile, western blotting analysis revealed that Y-27632 significantly increased the phosphorylation of ERK in the hHFSCs, which was mitigated upon treatment with U0126. These results indicate that Y-27632 maintained the proliferation and stemness of hHFSCs through the ERK/MAPK signaling pathway.

## Conclusion

We successfully established a xenofree short-term culture model for primary hHFSCs and demonstrated that the supplementation of Y-27632 in K-SFM promoted the adhesion, proliferation, stemness of hHFSCs and promoted the ability to regenerate hair follicles *in vivo*. Compared with the traditional method, the coating matrix and small molecule inhibitors used in the culture system could avoid the risk of heterogeneous gene contamination. These findings may aid in obtaining an increased number of high-quality seed cells, which have applications in hair follicle tissue engineering and stem cell therapy.

## Data Availability Statement

The original contributions presented in the study are included in the article/Supplementary Material, further inquiries can be directed to the corresponding author/s.

## Ethics Statement

The studies involving human participants were reviewed and approved by the Medical Ethical Committee of Southern Medical University. The patients/participants provided their written informed consent to participate in this study. The animal study was reviewed and approved by the Experimental Animal Centre at Southern Medical University.

## Author Contributions

LW, JZ, and YG performed all the experiments and prepared the figures and tables. ZF, DD, JH, and ZL provided with the statistical assistance. LW wrote the first draft of the manuscript. RC and YM revised the manuscript for important intellectual content. ZH and RC contributed to the conception and design of the study. All authors contributed to manuscript revision, read, and approved the submitted version.

## Conflict of Interest

The authors declare that the research was conducted in the absence of any commercial or financial relationships that could be construed as a potential conflict of interest.

## Supplementary Material

Supplementary Figure 1 Y-27632 promoted the effective passages on short-term culture of hHFSCs. Both hHFSCs with or without Y-27632 at early passages (P1-2) were actively dividing and appeared small, cuboidal, and homogeneous. However, less cells could reattached onto culture dishes and became senescence with the flat and heterogeneous morphology at later passages (P3-4) in the untreated group. The hHFSCs with Y-27632 can be continuously cultured for at least 5 passages with a healthy status. Unfortunately only approximately 50% of the treated cells escaped senescence at passage 6. Scale bars: 100 μm.
