# Supplementary figures and images for: Establishment of an Efficient Primary Culture System for Human Hair Follicle Stem Cells Using the Rho-Associated Protein Kinase Inhibitor Y-27632
Source: Front Cell Dev Biol. 2021 Mar 5;9:632882. doi: 10.3389/fcell.2021.632882 (PMC7973216; doi:10.3389/fcell.2021.632882)

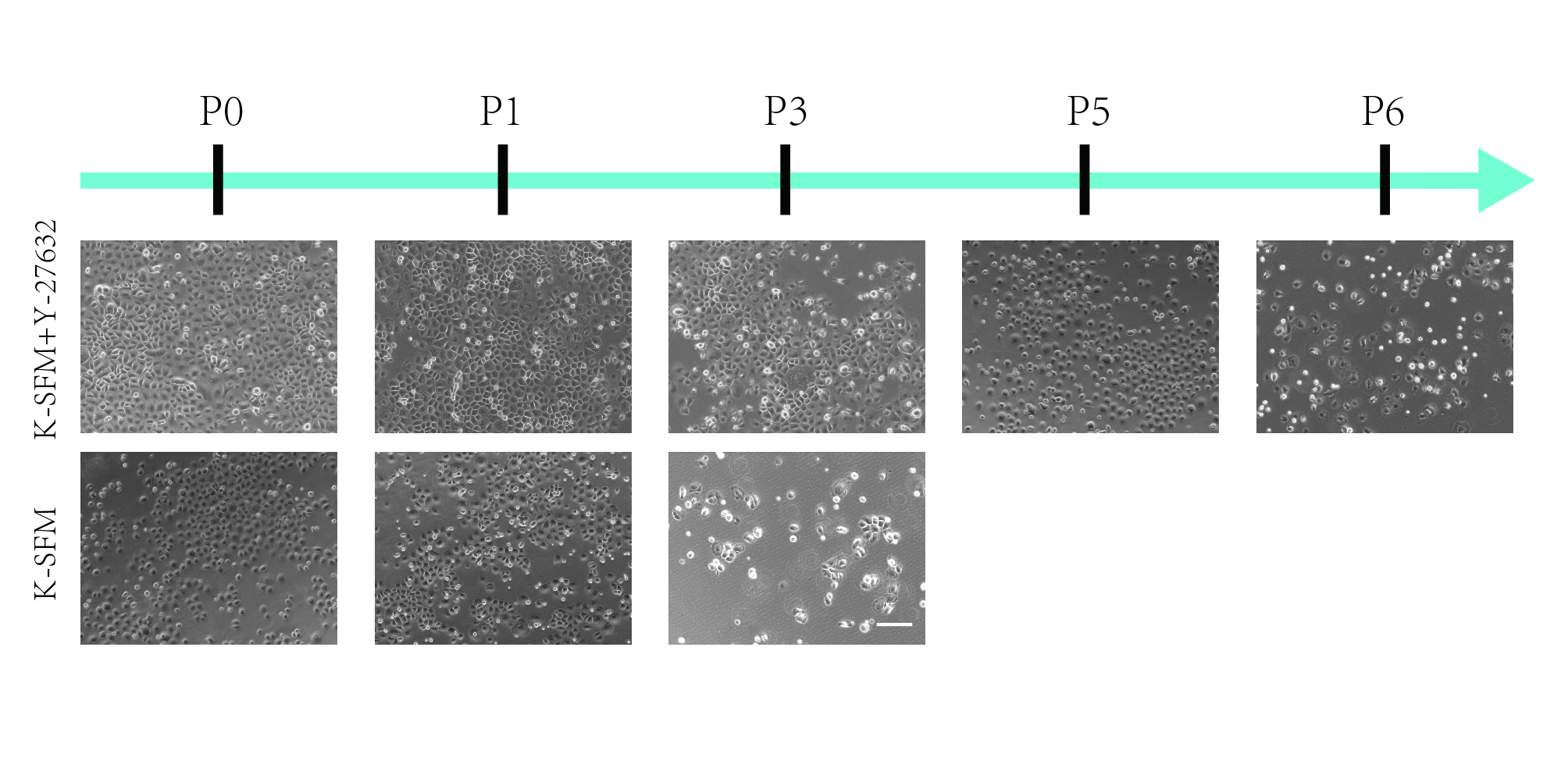

Supplement: Supplementary Figure 1 — Y-27632 promoted the effective passages on short-term culture of hHFSCs. Both hHFSCs with or without Y-27632 at early passages (P1-2) were actively dividing and appeared small, cuboidal, and homogeneous. However, less cells could reattached onto culture dishes and became senescence with the flat and heterogeneous morphology at later passages (P3-4) in the untreated group. The hHFSCs with Y-27632 can be continuously cultured for at least 5 passages with a healthy status. Unfortunately only approximately 50% of the treated cells escaped senescence at passage 6. Scale bars: 100 μm. [file Image_1.JPEG]
